# Validation of the Arabic Dementia Diagnosis Attitude Scale (A‐DDAS)

**DOI:** 10.1002/dad2.70262

**Published:** 2026-02-12

**Authors:** Nibras Jasim, Eman Shatnawi, Flor Sanabria Vasquez, Yousra Ali, Lyn Phillipson, Déborah Oliveira, Tiet‐Hanh Dao‐Tran, Genevieve Z. Steiner‐Lim, Diana Karamacoska

**Affiliations:** ^1^ School of Psychology Western Sydney University Sydney New South Wales Australia; ^2^ NICM Health Research Institute Western Sydney University Sydney New South Wales Australia; ^3^ The MARCS Institute for Brain Behaviour and Development, Western Sydney University Sydney New South Wales Australia; ^4^ School of Health and Society Faculty of Arts, University of Wollongong, Social Sciences and Humanities Wollongong New South Wales Australia; ^5^ Faculty of Nursing Campus Viña del Mar Universidad Andrés Bello Valparaíso Chile; ^6^ Centre for Health Services Research Faculty of Health, Medicine & Behavioural Sciences University of Queensland Brisbane Queensland Australia; ^7^ Department of Psychiatry and Psychotherapy Jena University Hospital Jena Germany

**Keywords:** Alzheimer's disease, beliefs, perceptions, psychometrics, stigma

## Abstract

**INTRODUCTION:**

Culturally appropriate scales are needed to efficiently assess stigma among Arabic‐speaking communities. This study aimed to validate the Arabic version of the Dementia Diagnosis Attitude Scale (A‐DDAS).

**METHODS:**

The translated A‐DDAS underwent pre‐testing with native speakers in Australia. The final version of the scale was tested with Arabic‐speaking adults aged ≥ 18 residing in Australia. The sample (*N *= 266) was randomly split such that one half (*n *= 133) underwent exploratory factor analysis and the other half (*n *= 133) underwent confirmatory factor analysis. Internal consistency reliability was assessed via Cronbach α.

**RESULTS:**

The final 10‐item scale consisted of two factors with five items each: “fear of labelling” (α = 0.88) and “fear of discrimination” (α = 0.85), with inter‐factor correlation *r *= 0.51 and high reliability (α = 0.87).

**DISCUSSION:**

The A‐DDAS yielded good validity and reliability scores, confirming its suitability for use with Arabic‐speaking Australians in stigma studies, educational interventions, and clinical settings.

## Highlights

- A culturally appropriate, valid, and reliable tool is needed to assess dementia attitudes among Arabic‐speaking populations.
- The Arabic version of the Dementia Diagnosis Attitude Scale (A‐DDAS) was administered with Arabic‐speaking Australians to assess its validity and reliability.
- The 10‐item A‐DDAS is valid and reliable for measuring the dementia attitudes of Arab‐speaking individuals in Australia. Further research is needed to validate this tool in diverse geographical contexts where Arabic is spoken.

## 1 BACKGROUND

Dementia is a neurodegenerative condition that causes progressive cognitive decline and impacts a person's ability to carry out activities of daily living.^1^ It affects > 55 million people worldwide, with nearly 10 million people diagnosed every year.^2^ More than 80% of people living with dementia worldwide report experiencing discrimination;^3^ therefore, it is urgent to better understand individual attitudes related to dementia among the general public so that effective anti‐stigma interventions can be developed and implemented to reduce discrimination at a global scale. This paper focus on Arabic‐speaking communities, a growing but under‐represented cohort in dementia research and services.

Within Arabic‐speaking cultures, people with dementia are typically regarded as being helpless, dependent, and deprived of their rights.^4^ These beliefs can lead to the internalization of negative stereotypes, prejudice, and discriminatory treatment.^3^ The medical term for dementia, *kharaf*, translates to “loss of mind” and is conflated with mental illness.^5^, ^6^, ^7^ Consequently, dementia is regarded as taboo and its diagnosis comes with social ramifications, such as isolation, and hinders people from gaining an early diagnosis, participating in research, and accessing care services.^8^, ^9^, ^10^

Across various Arabic‐speaking nations such as Jordan,^11^ Lebanon,^12^ and Saudi Arabia,^13^ misperceptions of dementia as a natural part of aging prevail. In Lebanon, dementia knowledge gaps and having an older age, lower socioeconomic level, and higher religiosity scores have been linked with higher levels of stigma toward people with dementia.^4^, ^12^ In the Arabic diaspora, negative cultural norms and stigmatizing views continue to be held, with one Australian study highlighting the persisting belief that the diagnosis should be concealed.^8^ These studies indicate an urgent need to address the stigma and misperceptions related to dementia among Arabic‐speaking communities, particularly in diasporas where pre‐existing sociocultural and language barriers further impede access to information and care provisions.^5^

Several awareness‐raising initiatives targeting Arabic‐speaking communities have commenced in Australia. The National Ageing Research Institute has produced a suite of online videos and comics in Arabic (movingpictures.org.au/Listing/Category/arabic) and Dementia Australia hosts a digital library of videos, audios, and fact sheets (dementia.org.au/languages/arabic). Our research group has co‐created online and face‐to‐face education sessions in partnership with a multicultural dementia advocacy group known as the Canterbury Bankstown Dementia Alliance.^5^, ^14^ However, measuring the efficacy of any public health, sociocultural, and/or educational intervention in reducing stigma requires a culturally appropriate, valid, and reliable tool. To the best of our knowledge, there are no measurement tools in the Arabic language to assess attitudes related to dementia. Within the existing studies conducted in Arab communities, Aljezawi et al.^11^ used the English version of O'Connor and McFadden's Dementia Attitudes Scale;^15^ and Alhazzani et al.^13^ forward translated the Alzheimer's Disease Knowledge Assessment Scale and inferred participants’ attitudes based on one item without validating the tool. Considering the current need for a validated scale to measure attitudes toward dementia, we aimed to translate and validate a dementia attitude scale that was originally developed for English‐speaking Australians.^16^ This scale has also been translated for use in Simplified Chinese^17^ and Japanese,^18^, ^19^ highlighting its cross‐cultural relevance in assessing stigma.

## 2 MATERIALS AND METHODS

This study was conducted in partnership with the Canterbury Bankstown Dementia Alliance to facilitate the evaluation of community‐based interventions targeting culturally and linguistically diverse people, including those that speak Arabic. The Alliance collaborated with the research team in all steps of the current study.

**RESEARCH IN CONTEXT**

1. **Systematic review**: Academic and gray literature concerning the negative attitudes held toward dementia among Arab nations and the Arabic‐speaking diaspora were reviewed. To the best of our knowledge, there are no measurement tools in the Arabic language that assess attitudes toward a dementia diagnosis.
2. **Interpretation**: We culturally adapted and validated a 10‐item dementia diagnosis attitude scale among Arabic‐speaking individuals in Australia.
3. **Future directions**: This newly developed scale is suitable for use in stigma studies, educational or public health interventions, and clinical settings. Future studies could review the applicability of this scale in migrant and non‐migrant contexts, given the paucity of research in this area.

### 2.1 Scale content

The lead author of the Australian Dementia Attitude Scale^16^ was contacted for permission to translate it into Arabic. The original scale consisted of 28 statements adapted from the Fraboni Scale of Ageism and the Perceptions Regarding Investigational Screening for Memory in Primary Care (PRISM‐PC)–Dementia Screening Subscale. The Dementia Alliance was consulted about the relevance of the content of the Phillipson et al.’s 28‐item survey, ^16^ collectively agreeing that it was too long for the cultural communities they represent, recommending the removal of the first 16 items and focusing on the 12 items concerning a person's feelings, perceptions, and behaviors toward a dementia diagnosis. Each item requires a response on a five‐point Likert scale (“strongly agree” to “strongly disagree”) to rate 12 items beginning with the statement “If I were diagnosed with dementia….” As reported by Phillipson et al., ^16^ the first eight items probing feelings of shame, stupidity, embarrassment, depression, and anxiety associated with a dementia diagnosis (e.g., “I would feel depressed” and “I would not want my family to know”) were associated with the factor “fear of labelling.” The remaining four items concerning adequate medical treatment, health insurance, and employment were associated with the second factor, “fear of discrimination.”

The present scale is referred to as the Arabic version of the Dementia Diagnosis Attitude Scale (A‐DDAS) and was collated into a survey containing additional demographic questions (age, sex, country of birth, education level, employment status, and whether participants knew someone living with dementia) to characterize our study sample.

### 2.2 Scale translation

All research materials (including the participant information sheet, advertising material, and survey) were forward translated from English to Arabic by an accredited translator. Due to funding constraints, one Arabic translator (accredited by the National Accreditation Authority for Translators and Interpreters) independent to the research team was engaged to forward translate the survey comprising the scale. The translated version of the scale was reviewed by the Arabic‐speaking community members associated with the Alliance (*n *= 7), as well as by two bilingual researchers (E.S., N.J.), to identify issues related to comprehension and relevance with the goal of enhancing face validity. Each scale item was reviewed one‐by‐one with requests for substitutions or revisions discussed and recorded with the bilingual researchers. Members were hesitant about the use of the medical translation for dementia (*kharaf*) but deemed this necessary given its continued application in Australian‐based Arabic information resources and health‐care settings, and lack of an alternative term, reinforcing the need for stigma‐reduction campaigns. Based on our community members’ recommendations, advertising materials did not use this terminology and instead referred to brain health. In addition, they recommended including a preamble to the survey to reiterate the scale's purpose and characterization of dementia. Concerns were raised about two scale items (“I would feel humiliated” and “I would give up on life”) as there were no direct translations of these into Arabic and were deemed to be unaligned with traditional cultural values of the Arabic community. It was thus decided that these two items would be removed from the Arabic scale. Another item's literal translation required revision to better contextualize its translated meaning: “I would be considered stupid and unable to do *things*” was revised to “I would be considered stupid and unable to do *tasks*.” The revised survey was returned to the accredited translator for back translation into English and the research team (comprising bilingual researchers [E.S., N.J.] and content experts [D.K., G.Z.S., and D.O.]) checked this for comparability to the original version. No further changes were required as the bilingual researchers asserted that the meaning and consistency of scale items were retained. The final version of the survey (including the demographic and scale items) was hosted digitally on the Qualtrics platform and a copy can be found in the supporting information (in English and Arabic). Ethical approval for this study was obtained from the Western Sydney Human Research Ethics Committee (reference H15442).

### 2.3 Participant recruitment

Adults aged ≥ 18 who lived in Australia and could read the Arabic language were recruited using a snowballing technique. We used social media posts on Facebook and LinkedIn targeting Arabic‐speaking groups for migrant Arabic Australians and face‐to‐face visits to Sydney‐based social groups where hard copies of the Arabic survey were disseminated. Access to these groups was facilitated through local health and welfare service providers who were contacted through the Alliance by bilingual researchers. A brief explanation of the survey's purpose, timing, benefits, risks of discomfort, and study staff's contact details was shared through a participant information sheet that individuals received and read before starting the survey. Participants were encouraged to take as long as they wished to complete the survey. Consent was implied by completing the survey with the option of entering a draw to win $200 after survey completion. Surveys completed in hardcopy format were typed into Qualtrics by bilingual researchers. Data were collected during May to December 2023.

### 2.4 Data analysis

As per the Watkin sample size guide,^20^ at least 20 participants per scale item were required for exploratory and confirmatory factor analysis (*N* = 200). Survey data were exported from Qualtrics into Excel and translated by bilingual researchers from Arabic to English for analysis. Incomplete scale data from 47 individuals were removed, resulting in a sample of 266 participants for complete case analysis. Statistical analyses were carried out in SPSS (version 27.0). Demographic data were analyzed descriptively. The dataset was randomly split in half using the select cases’ function in SPSS to undertake exploratory factor analysis (EFA) (*n* = 133) and confirmatory factor analysis (CFA; *n* = 133). Sample adequacy was checked via the Kaiser–Meyer–Olkin (KMO) score (≥ 0.60) and the Bartlett test of sphericity (*P *≤ 0.05).^20^, ^21^ Decisions regarding item retention were based on communality scores (> 0.33), pattern matrix loading scores (≥ 0.4), and item‐total correlation (≥ 0.3).^22^ Principal axis factoring analysis with Promax rotation with 25 iterations for convergence was used to extract factors that met the following criteria: eigenvalue > 1, the visualization of a Cattell scree plot, and inter‐factor correlations (0.3–0.7).^22^ Internal consistency was assessed using Cronbach alpha coefficient (α ≥ 0.7 indicated good reliability) and this was done for each factor and the overall scale.^23^ Retained items and factors were reviewed by the bilingual researchers to ensure appropriate and meaningful interpretation.

Analysis of Moment Structures (AMOS; version 29.0) was used to perform CFA on the second half of participants (*n *= 133). Goodness of fit was achieved with two or more of the following criteria: the weighted χ^2^/df  was < 3, cumulative fit index (CFI) was > 0.90, Tucker–Lewis Index (TLI) > .90, standardized root mean square residual (SRMR) < 0.08, root mean square error of approximation (RMSEA) was < 0.10, and the cut‐off for item loading onto a factor was at least 0.30.^24^, ^25^, ^26^, ^27^, ^28^, ^29^

## 3 RESULTS

### 3.1 Participant characteristics

The 266 participants were aged between 18 and 84 years old (mean = 45.1, standard deviation = 13.5), 84.6% of whom were women (*n* = 225). Most were born in Iraq (49.6%), had a university level of school attainment (57.9%), and did not know someone living with dementia (53.0%; Table 1).

**TABLE 1:** Participant characteristics (N = 266).

Variable	*n* (%)
Sex	
Male	38 (14.3)
Female	225 (84.6)
Prefer not to say/no response	3 (1.2)
Birthplace	
Iraq	132 (49.6)
Syria	29 (10.9)
Lebanon	17 (6.4)
Egypt	7 (2.6)
Australia	4 (1.5)
Jordan	4 (1.5)
Kuwait	3 (1.1)
Palestine	2 (0.8)
Africa	1 (0.4)
Algeria	1 (0.4)
Israel	1 (0.4)
Libya	1 (0.4)
Qatar	1 (0.4)
Saudi Arabia	1 (0.4)
Sudan	1 (0.4)
Tunisia	1 (0.4)
No response	60 (22.5)
Employment	
Unemployed/not working	101 (38.0)
Caregiving	52 (19.5)
Retired	30 (11.3)
Working	81 (30.5)
No response	2 (0.8)
School attainment	
Primary/secondary schooling	88 (33.1)
Trade/another certificate	31 (11.7)
University	143 (53.8)
No response	4 (1.5)
Know someone with Alzheimer's disease or dementia	
Yes	124 (46.6)
No	141 (53.0)
No response	1 (0.4)

### 3.2 Exploratory factor analysis

No multicollinearity was identified (inter‐item correlations ranged *r* .159–.799) and data appeared to be suitable for factor analysis (KMO = 0.81; Barlett test: *P *< 0.001). Initial item‐total correlations were all ≥ 0.419 (Table S1 in supporting information).

Two factors were extracted and explained 65.4% of the total variance, and all 10 items met the retention criteria within this two‐factor solution (Tables 2 and 3). The first factor comprised the first five items (2–6) that distinctly associated a person's feelings and thoughts about being diagnosed with dementia, in line with the original study's “fear of labelling” subscale. The second factor contained the remaining five items (8–12) that reflected a person's concerns with disclosing their diagnosis to others and was designated “fear of discrimination.” The inter‐factor correlation was acceptable (*r *= 0.51).

**TABLE 2:** Communalities scores.

Item	
If I were diagnosed with dementia…	Communalities
1. I would feel humiliated	Removed
2. I would no longer be taken seriously	0.541
3. I would be considered stupid and unable to do tasks	0.495
4. I would be ashamed or embarrassed	0.749
5. I would be depressed	0.659
6. I would be anxious	0.520
7. I would give up on life	Removed
8. My doctor would not provide the best care for my other medical problems	0.516
9. My doctor and other health professionals would not listen to me	0.604
10. I would not want my health insurance company to find out	0.571
11. I would not want my employer to find out	0.544
12. I would not want my family to know	0.494

*Note*: Items 1 and 7 were removed to enhance the scale's face validity.

**TABLE 3:** Pattern matrix scores, eigenvalues and variance explained for the 10‐item Arabic version of the Dementia Diagnosis Attitude Scale.

If I were diagnosed with dementia…	Factor 1: Fear of labelling	Factor 2: Fear of discrimination
2. I would no longer be taken seriously إذا تم تشخيصي بالخرف… فلن يتم أخذي على محمل الجد بعد الآن	0.724	
3. I would be considered stupid and unable to do tasks إذا تم تشخيصي بالخرف… فسيتم اعتباري غبيًا وغير قادر على القيام بالأشياء	0.699	
4. I would be ashamed or embarrassed إذا تم تشخيصي بالخرف… سأشعر بالخجل أو الإحراج	0.851	
5. I would be depressed إذا تم تشخيصي بالخرف… سأشعر بالاكتئاب	0.805	
6. I would be anxious إذا تم تشخيصي بالخرف… سأشعر بالتوتر والقلق	0.746	
8. My doctor would not provide the best care for my other medical problems إذا تم تشخيصي بالخرف… لن يقدم طبيبي أفضل رعاية لمشاكلي الطبية الأخرى		0.689
9. My doctor and other health professionals would not listen to me إذا تم تشخيصي بالخرف… لن يستمع لي طبيبي وغيره من مهنيي الصحة		0.784
10. I would not want my health insurance company to find out إذا تم تشخيصي بالخرف… لن أرغب في أن تكتشف وتعرف بذلك شركة التأمين الصحي خاصتي		0.759
11. I would not want my employer to find out إذا تم تشخيصي بالخرف… لا أريد أن يكتشف رب عملي ذلك		0.744
12. I would not want my family to know إذا تم تشخيصي بالخرف… لا أريد أن تعرف عائلتي بذلك		0.704
Eigenvalue	4.73	1.81
Variance explained (%)	47.30	18.14
Total variance explained (%)	65.44	

### 3.3 Confirmatory factor analysis

The CFA indicated an acceptable fit. Fit indices were mixed: the CFI = 0.95 suggested good fit, and the χ^2^/df = 2.21 indicated acceptable model fit. The TLI = 0.92 and the SRMR = 0.08 were within or close to conventional cutoffs for adequacy. The RMSEA = 0.096 (90% confidence interval: 0.07–0.13) suggested marginal fit, indicating slight model misfit. Overall, considering all indices, the model demonstrates a reasonable but not perfect fit.

**FIGURE 1:**
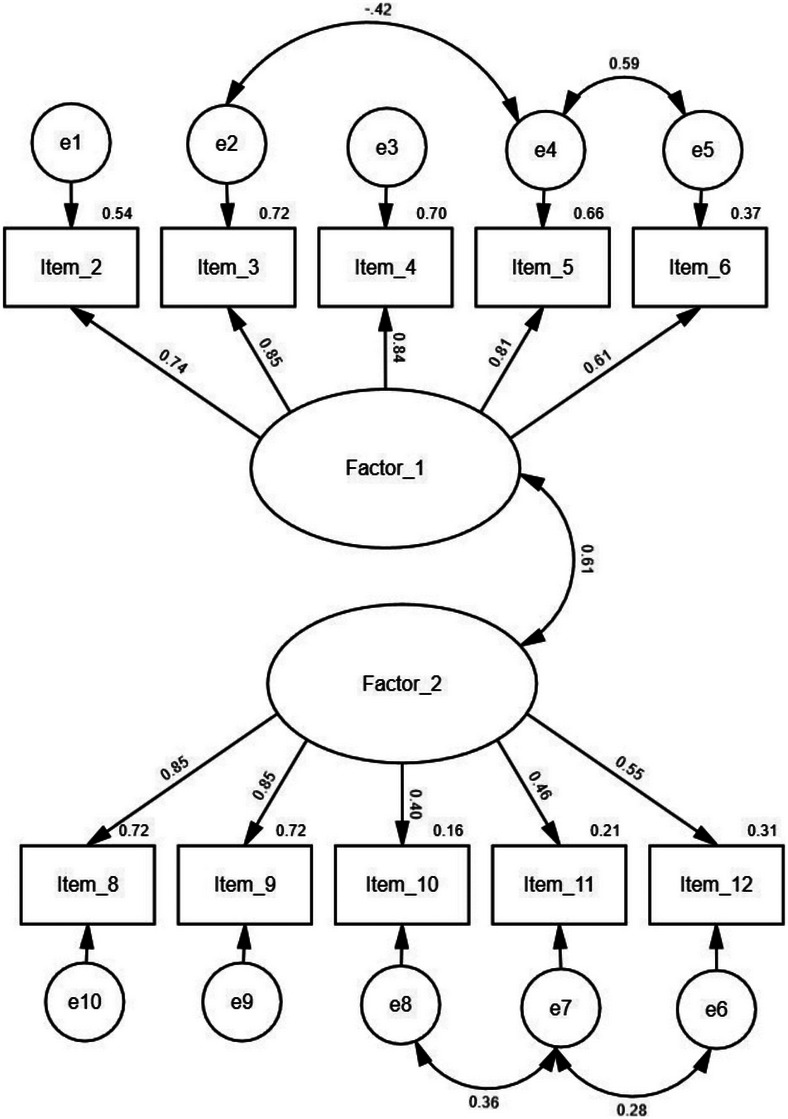
Factor loadings, inter‐factor correlations, and measurement errors (e) for the two‐factor solution. Factor 1 = Fear of labelling; Factor 2 = Fear of discrimination.

Factor 1 (fear of labelling) included items 2, 3, 4, 5, and 6. Factor 2 (fear of discrimination) included items 8, 9, 10, 11, and 12. Except for items 6, 10, 11, and 12, which had factor loadings of 0.40 to 0.61, all other items had at least 0.70. There were significant correlations between the residuals of item 3 (“If I were diagnosed with dementia… I would be considered stupid and unable to do tasks”) and item 5 (“If I were diagnosed with dementia, I would be depressed”); as well as item 5 and item 6 (“If I were diagnosed with dementia, I would be anxious”); items 10 (“If I were diagnosed with dementia, I would not want my health insurance company to find out)” and 11 (“If I were diagnosed with dementia, I would not want my employer to find out”); and items 11 and 12 (“If I were diagnosed with dementia, I would not want my family to find out”). The theoretical rationale for allowing these correlations is that the items share overlapping content (negative self‐image or not ready to disclose the diagnosis to others). Modification indices and residuals supported including these correlated errors to improve model fit. By explicitly specifying these residual correlations in the model, we ensured that the model fit reflects both the latent factor structure and theoretically justified shared variance among items. The correlation between the two factors was moderate (Figure 1).

### 3.4 Internal consistency

The general reliability of the 10‐item solution and for each of the two factors was excellent (general α = 0.87; factor 1 α = 0.88; factor 2 α = 0.85.).

## 4 DISCUSSION

We have reported the validation of the A‐DDAS for use within Arabic‐speaking communities in Australia. A 10‐item solution containing 10 items and two factors indicated satisfactory construct validity and high internal consistency. These outcomes are comparable to the existing English, Japanese, and Simplified Chinese versions of the scale,^16^, ^17^, ^18^, ^19^ with item‐specific differences reflecting nuances between cultures.

The cultural adaptation of this scale with community members resulted in two items being removed. The item probing humiliation was considered redundant due to translation issues. Humiliation is an umbrella term for multiple losses including feelings of sadness, despair, and loss of hope,^30^ with a lack of direct translation. The second item related to “giving up on life” after a dementia diagnosis is against the Arabic culture's emphasis on sustaining life and well‐being through familial care no matter the circumstances.^8^, ^31^, ^32^, ^33^, ^34^, ^35^ In the Arabic culture, it is expected that immediate and extended family members provide care for family members experiencing illness, a practice rooted in both social and religious obligations.^36^

The 10‐item scale yielded two factors with strong item loadings (≥ 704), the first of which corresponded with the original “fear of labelling” identified in prior studies.^16^, ^17^, ^18^ This factor also aligns with the Arabic literature highlighting the concerns associated with a dementia diagnosis, such as the person not being taken seriously by other people and considered unable to do basic tasks.^4^, ^8^ It also validated the anticipated feelings of embarrassment, depression, and anxiety associated with having dementia.^8^, ^13^ The correlations among items 3, 5, and 6 (respectively assessing perceptions of being considered stupid, depression, and anxiety) suggested the potential for experiencing mixed mental health conditions when being diagnosed with dementia.^4^, ^37^ This internalization of negative feelings may underscore the stigma associated with help‐seeking, requiring psychoeducational support to navigate the diagnostic process.^38^ Additionally, the correlations among items 10 (not wanting my health insurance company to find out), 11 (not wanting my employer to find out), and 12 (not wanting my family to find out) coincide with the ramifications one may experience in disclosing a dementia diagnosis to various circles or institutions (reduced social standing, increased sick leave, and reduced income to name a few^39^, ^40^). It must also be recognized that scale items concerning insurance and employment were specific to the Australian context and their applicability in other Arabic‐speaking communities and nations may differ. Future research should consider contextual re‐validation and to review the applicability of this scale in migrant and non‐migrant contexts given the paucity of research in this area.^8^

Interestingly, item 12 (disclosing the diagnosis to family) loaded onto the second factor in the A‐DDAS as opposed to the first factor from the original English scale.^16^ As this item was clustered with other items concerning the implications of disclosing their diagnosis in health‐care and workplace contexts, this second factor was regarded as “fear of discrimination.” This means that the Arabic‐speaking Australians may anticipate stigma with a dementia diagnosis in a variety of contexts including their family.^8^ This factor was similarly observed in the Simplified Chinese version of the DDAS.^17^ Collectivist values in Chinese‐ and Arabic‐speaking cultures intersect with family honor and one's social standing. Disclosing a diagnosis of a disability like dementia can have major consequences on individuals, their families, one's reputation, societal roles, and religious adherence.^8^, ^9^, ^10^ Deeper explorations into the drivers of these stigmatic attitudes can be supplemented with a future study involving mixed methods or qualitative responses to the scale items.

Although the study provided support for the structural validation of the scale with Arabic‐speaking populations, some limitations highlight the need for further research. While maximum likelihood estimation in AMOS is widely used for 5‐point Likert data, alternative estimators (e.g., Weighted Least Squares Mean and Variance adjusted or WLSMV in Mplus or lavaan) may provide a more optimal treatment of ordinal variables. Participants were also disproportionately composed of women (84%) and from a highly educated background, which may introduce sex‐ and education‐related biases, limiting the generalizability of this study. The present study may not reflect the attitudes of Arabic‐speaking individuals with lower education levels.^12^, ^13^ Women in the community are also more likely to be caregivers of someone with dementia, which may impact their attitudes toward dementia.^8^ Further, it is well documented that dementia is considered a taboo topic in these communities, which may have contributed to lower participation rates among those with varying education levels and those with higher levels of stigma.^8^ Future studies should aim to recruit more diverse and balanced samples to ensure the generalizability of the findings and use methods such as weighting, sensitivity analyses, or item response theory to explore measurement invariance and assess the robustness of the instrument within different population subgroups.

Our sampling limitations also highlight the need for inclusive research that uses strategic recruitment, promotion, and engagement of participants. As advised by the Canterbury Bankstown Dementia Alliance and affiliated community members, advertisements could avoid using the translated term for dementia and instead refer to “brain health,” “memory changes or loss,” or “Alzheimer's disease.”^5^ Using interpreters or bilingual researchers to collect data using telephone or videoconferencing services would facilitate a more sensitive approach to such research, during which participants can be eased into the topic of dementia and questions can be addressed in real time.^41^ A vignette or case study of a person's experience with dementia can also be used to engage responses to the survey (see Phillipson et al.^42^). Finally, funding availability constrained independent translation efforts. To overcome this, we prioritized input from both bilingual researchers and native‐speaking community members to ensure the translated survey and its contents remained relevant to the targeted population. Future studies should adequately resource their scale translation and validation efforts according to best‐practice guidelines.^43^, ^44^

Understanding the Arabic‐speaking community's attitudes toward dementia is important to researchers, policy makers, and care providers in realizing several national and global dementia plan actions. The present study provided evidence for the A‐DDAS's structural validity; however, future studies should adopt additional psychometric tests to examine construct validity (convergent, divergent, discriminant validity), predictive validity, responsiveness, and test–retest reliability after a set interval to establish stronger validity and reliability evidence.^44^ The 10‐item A‐DDAS may thus serve as a helpful tool for exploring dementia attitudes in a variety of settings, including population‐level assessments (e.g., surveying the public about their attitudes to inform service planning), measuring changes pre‐to‐post intervention, and in clinical contexts as part of an initial brain health consult or assessment to determine information, resource, and service provision.

## CONFLICT OF INTEREST STATEMENT

D.K. and G.Z.S. are academic researchers at NICM Health Research Institute who are members of the Canterbury Bankstown Dementia Alliance but have no conflicts of interest to declare. As a medical research institute, NICM Health Research Institute receives research grants and donations from foundations, universities, government agencies, individuals, and industry. Sponsors and donors provide untied funding for work to advance the vision and mission of the institute. All other authors have no conflicts of interest to declare. disclosures are available in the supporting information

## CONSENT STATEMENT

Implied consent was obtained from all human participants who completed the survey research. Ethical approval for the conduct of this research was provided by Western Sydney University Human Research Ethics Committee (reference H15442).

## Supporting information



Supporting Information

Supporting Information
