# Supplementary material for: Validation of the Arabic Dementia Diagnosis Attitude Scale (A‐DDAS)
Source: Alzheimers Dement (Amst). 2026 Feb 12;18(1):e70262. doi: 10.1002/dad2.70262 (PMC12897567; doi:10.1002/dad2.70262)
Supplement: Supplementary file 1 — Supporting Information [file DAD2-18-e70262-s002.docx]

**Supplementary Materials**

**English version of the Dementia Diagnosis Attitude Scale**

**PREAMBLE**

| This survey explores the topic of Alzheimer’s disease, which is a brain illness that causes memory loss. It is the most common type of dementia. Dementia is a medical term that refers to a collection of diseases and disorders that cause memory loss and behaviour changes that affect everyday life.    Please read the accompanying Participant Information Sheet before starting this survey. By completing this survey, you are providing implied consent for your participation in this research. Participation is voluntary. If you have any questions, please contact the researchers named on the attached Participant Information Sheet. |
| --- |

Using the below scale, rate how much you disagree or agree with each statement:

**1. If I were diagnosed with dementia… I would feel humiliated**

| - Strongly disagree | - Disagree | - Neutral | - Agree | - Strongly agree |
| --- | --- | --- | --- | --- |

**2. If I were diagnosed with dementia… I would no longer be taken seriously**

| - Strongly disagree | - Disagree | - Neutral | - Agree | - Strongly agree |
| --- | --- | --- | --- | --- |

**3. If I were diagnosed with dementia… I would be considered stupid and unable to do things**

| - Strongly disagree | - Disagree | - Neutral | - Agree | - Strongly agree |
| --- | --- | --- | --- | --- |

**4. If I were diagnosed with dementia… I would be ashamed or embarrassed**

| - Strongly disagree | - Disagree | - Neutral | - Agree | - Strongly agree |
| --- | --- | --- | --- | --- |

**5. If I were diagnosed with dementia… I would be depressed**

| - Strongly disagree | - Disagree | - Neutral | - Agree | - Strongly agree |
| --- | --- | --- | --- | --- |

**6. If I were Diagnosed with Dementia… I would be anxious**

| - Strongly disagree | - Disagree | - Neutral | - Agree | - Strongly agree |
| --- | --- | --- | --- | --- |

**7. If I were diagnosed with dementia… I would give up on life**

| - Strongly disagree | - Disagree | - Neutral | - Agree | - Strongly agree |
| --- | --- | --- | --- | --- |

**8. If I were diagnosed with dementia… My doctor would not provide the best care for my other medical problems**

| - Strongly disagree | - Disagree | - Neutral | - Agree | - Strongly agree |
| --- | --- | --- | --- | --- |

**9. If I were diagnosed with dementia… My doctor and other health professionals would not listen to me**

| - Strongly disagree | - Disagree | - Neutral | - Agree | - Strongly agree |
| --- | --- | --- | --- | --- |

**10. If I were diagnosed with dementia… I would not want my health insurance company to find out**

| - Strongly disagree | - Disagree | - Neutral | - Agree | - Strongly agree |
| --- | --- | --- | --- | --- |

**11. If I were diagnosed with dementia… I would not want my employer to find out**

| - Strongly disagree | - Disagree | - Neutral | - Agree | - Strongly agree |
| --- | --- | --- | --- | --- |

**12. If I were diagnosed with dementia… I would not want my family to know**

| - Strongly disagree | - Disagree | - Neutral | - Agree | - Strongly agree |
| --- | --- | --- | --- | --- |

**Arabic Dementia Diagnosis Attitude Scale (A-DDAS)**

**المقدمة**

**
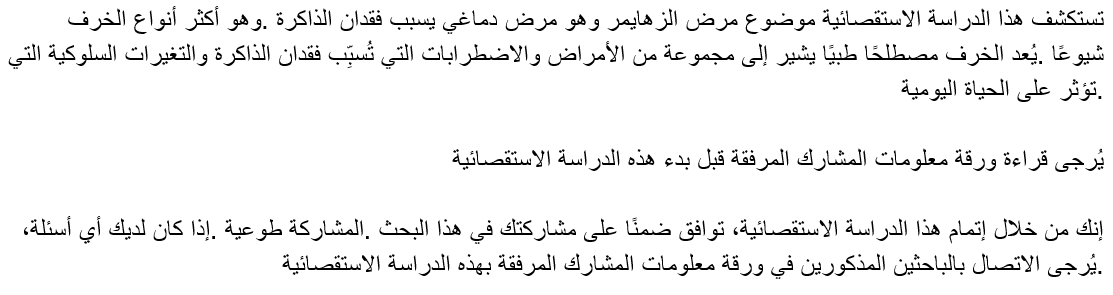
**

باستخدام المقياس أدناه، قيِّم مدى معارضتك أو موافقتك لكل عبارة/إفادة:

**إذا تم تشخيصي بالخرف... سأشعر بالإهانة**

| - أعارض بشدة | - أعارض | - محايد | - أوافق | - أوافق بشدّة |
| --- | --- | --- | --- | --- |

**إذا تم تشخيصي بالخرف... فلن يتم أخذي على محمل الجد بعد الآن**

| - أعارض بشدة | - أعارض | - محايد | - أوافق | - أوافق بشدّة |
| --- | --- | --- | --- | --- |

**إذا تم تشخيصي بالخرف... فسيتم اعتباري غبيًا وغير قادر على القيام بالمهام**

| - أعارض بشدة | - أعارض | - محايد | - أوافق | - أوافق بشدّة |
| --- | --- | --- | --- | --- |

**إذا تم تشخيصي بالخرف... سأشعر بالخجل أو الإحراج**

| - أعارض بشدة | - أعارض | - محايد | - أوافق | - أوافق بشدّة |
| --- | --- | --- | --- | --- |

**إذا تم تشخيصي بالخرف... سأشعر بالاكتئاب**

| - أعارض بشدة | - أعارض | - محايد | - أوافق | - أوافق بشدّة |
| --- | --- | --- | --- | --- |

**إذا تم تشخيصي بالخرف... سأشعر بالتوتر والقلق**

| - أعارض بشدة | - أعارض | - محايد | - أوافق | - أوافق بشدّة |
| --- | --- | --- | --- | --- |

**إذا تم تشخيصي بالخرف... سأستسلم وأتخلى عن الحياة**

| - أعارض بشدة | - أعارض | - محايد | - أوافق | - أوافق بشدّة |
| --- | --- | --- | --- | --- |

باستخدام المقياس أدناه، قيِّم مدى معارضتك أو موافقتك لكل عبارة/إفادة:

**إذا تم تشخيصي بالخرف... لن يقدم طبيبي أفضل رعاية لمشاكلي الطبية الأخرى**

| - أعارض بشدة | - أعارض | - محايد | - أوافق | - أوافق بشدّة |
| --- | --- | --- | --- | --- |

**إذا تم تشخيصي بالخرف... لن يستمع لي طبيبي وغيره من مهنيي الصحة**

| - أعارض بشدة | - أعارض | - محايد | - أوافق | - أوافق بشدّة |
| --- | --- | --- | --- | --- |

**إذا تم تشخيصي بالخرف... لن أرغب في أن تكتشف وتعرف بذلك شركة التأمين الصحي خاصتي**

| - أعارض بشدة | - أعارض | - محايد | - أوافق | - أوافق بشدّة |
| --- | --- | --- | --- | --- |

**إذا تم تشخيصي بالخرف... لا أريد أن يكتشف رب عملي ذلك**

| - أعارض بشدة | - أعارض | - محايد | - أوافق | - أوافق بشدّة |
| --- | --- | --- | --- | --- |

**إذا تم تشخيصي بالخرف... لا أريد أن تعرف عائلتي بذلك**

| - أعارض بشدة | - أعارض | - محايد | - أوافق | - أوافق بشدّة |
| --- | --- | --- | --- | --- |

**Note: greyed items were removed from the final validation version of the A-DDAS.**

**Supplementary Exploratory Factor Analysis Outcomes**

**Supplementary Table S1**

*Item-Total correlations for 10-Item Arabic DDAS*

| Item | Item-Total Correlations | |
| --- | --- | --- |
| If I were diagnosed with dementia… | |  |
| 1. I would feel humiliated | | Removed |
| 1. I would no longer be taken seriously | | .619 |
| 1. I would be considered stupid and unable to do tasks | | .580 |
| 1. I would be ashamed or embarrassed | | .718 |
| 1. I would be depressed | | .673 |
| 1. I would be anxious | | .568 |
| 1. I would give up on life | | Removed |
| 1. My doctor would not provide the best care for my other medical problems | | .573 |
| 1. My doctor and other health professionals would not listen to me | | .580 |
| 1. I would not want my health insurance company to find out | | .575 |
| 1. I would not want my employer to find out | | .553 |
| 1. I would not want my family to know | | .533 |
| Total Cronbach’s α | | .87 |
